# Histology-Verified Intracranial Artery Calcification and Its Clinical Relevance With Cerebrovascular Disease

**DOI:** 10.3389/fneur.2021.789035

**Published:** 2022-01-24

**Authors:** Heng Du, Wenjie Yang, Xiangyan Chen

**Affiliations:** ^1^Department of Health Technology and Informatics, The Hong Kong Polytechnic University, Kowloon, Hong Kong SAR, China; ^2^Department of Diagnostic Radiology and Nuclear Medicine, School of Medicine, University of Maryland, Baltimore, MD, United States

**Keywords:** intracranial artery calcification, histology, imaging, clinical relevance, cerebrovascular disease

## Abstract

Intracranial artery calcification (IAC) was regarded as a proxy for intracranial atherosclerosis (ICAS). IAC could be easily detected on routine computer tomography (CT), which was neglected by clinicians in the previous years. The evolution of advanced imaging technologies, especially vessel wall scanning using high resolution-magnetic resonance imaging (HR-MRI), has aroused the interest of researchers to further explore the characteristics and clinical impacts of IAC. Recent histological evidence acquired from the human cerebral artery specimens demonstrated that IAC could mainly involve two layers: the intima and the media. Accumulating evidence from histological and clinical imaging studies verified that intimal calcification is more associated with ICAS, while medial calcification, especially the internal elastic lamina, contributes to arterial stiffness rather than ICAS. Considering the highly improved abilities of novel imaging technologies in differentiating intimal and medial calcification within the large intracranial arteries, this review aimed to describe the histological and imaging features of two types of IAC, as well as the risk factors, the hemodynamic influences, and other clinical impacts of IAC occurring in intimal or media layers.

## Introduction

Calcification is widely located in all vascular beds (1, 2), especially in the advanced stages of atherosclerosis along with intraplaque hemorrhage, hemosiderin deposition, and lumen surface disruption (3). Over the last decades, significant progress has been made in clinical research on intracranial artery calcification (IAC). IAC in the intracranial internal carotid artery (ICA) was demonstrated as an independent risk factor for ischemic stroke which accounted for up to 75% of all strokes (4). Currently, most studies on IAC are based on computer tomography (CT) which is capable of providing overall views of single or multiple calcifications. Calcification score and volume (5), which were initially used for assessing coronary arteries, are now widely applied to qualitative and quantitative measurements in exploring the clinical relevance of IAC (6). Despite substantial research on IAC, the correlation of IAC with stroke is controversial. In the Rotterdam study, many of the ischemic strokes were either in the vascular territories that were separate from IAC or caused by other conditions, for instance, cardiac source embolism or by the coexisting penetrating artery diseases (4). Furthermore, despite the association of IAC and intraplaque hemorrhage (7), calcified atherosclerotic plaques in the middle cerebral arteries (MCAs) seemed to be more stable than the non-calcified plaques (8). The pathophysiology of IAC and stroke remains unclear.

There are two major patterns of IAC, one involving the intima and the other involving the media. Recent histopathologic evidence showed that medial calcification was predominantly present in both the intracranial internal carotid arteries (ICAs) (9) and vertebral arteries (VAs) (10), bringing about new considerations on the importance of IAC patterns in further clinical studies. In this review, we intended to discuss the histopathological features of IAC, its manifestation in CT and MRI as well as its clinical relevance, which may benefit in developing a better understanding of calcification and related diseases.

## Methods

Literature searching was performed in PubMed, with a search filter using words, such as “intracranial artery calcification,” “intimal calcification,” “medial calcification,” and “stroke.” All articles were extracted according to title and abstract. Most of the articles were original clinical studies. The full texts of the relevant articles were assessed independently. The references of the relevant articles were selected additionally for further evidence. Three hundred and sixty-eight articles about either the intracranial artery or the extracranial artery were screened. Articles based on autopsies, CT, and magnetic resonance imaging (MRI) were included. Articles focused on biochemical or genetic studies were excluded.

For studies based on autopsies, the identification of intimal or medial calcification in the intracranial artery was based on pathological evidence. Calcifications were identified as sharply demarcated, acellular spots, and areas. Calcification type was determined by adding the calcification areas in all slides to a summed intimal and medial calcification burden. If the summed area of medial calcification was larger than the summed area of intimal calcification, the patient was categorized by histology as a medial dominant and vice versa.

For studies based on brain CT, the definition of intimal or medial calcification in the intracranial artery was based on circularity (1 for dot, 2 for <90°, 3 for 90–270°, and 4 for 270–360°), thickness (1 for thick IAC ≥1.5 mm and 3 for thin IAC <1.5 mm), and morphology (0 for indistinguishable, 1 for irregular/patchy and 4 for continuous) on brain CT. A summed score from 1 to 6 indicated predominant intimal calcification and 7 to 11 indicated predominant medial calcification. For studies based on MRI, the identification of IAC pattern is yet to be studied.

## Imaging Measurement on IAC and the Existing Defects

Intracranial artery calcification is widely detected by brain CT scan due to its accessibility and reliability (11). In order to achieve quantitative analysis, semiautomatic custom-made methods are used *via* software, such as ImageJ (12) or MATLAB (13). The “volume” of IAC is calculated by multiplying the number, size, and the increment of pixels. Agatston score (14), which represents the area of calcified plaque multiplied by the weighted value assigned to its highest Hounsfield unit, has also been applied in the studies. Deficiently, Agatston score and calcium volume are both in demand for the slice thickness of 3 mm and for images without gantry tilts (6), and both are time-consuming and sometimes inaccessible for neurologists during clinical practice. In contrast, visual grading systems are comparatively more convenient for assessments. According to the previous grading criteria, the score increases as calcification extends either in the thickness or in the circumference (15–17).

However, since IAC embodied in the intima and media of intracranial arteries can vary in morphology and prevalence, the misleading effect of the Agatston score or the overall volume should not be neglected. In 2017, a new grading system was put forward by Kockelkoren et al. (18) which distinguished the intimal and medial calcification after comparing the histology and CT features of IAC. Circularity, thickness, and morphology were counted separately (Table 1) based on the distinct features of intimal and medial calcification. Of note, the grading order of thickness was conversed (“thick” represents one point and “thin” represents three points), and morphology was added into counting compared to prior grading systems (19).

**Table 1:** Scoring system [by Kockelkoren et al. (18)] for distinguishing intimal calcification from medial calcification.

**Characteristic: circularity, thickness, and morphology**		**Points**
Circularity	Absent	0
	Dot(s)	1
	<90 degrees	2
	96–270 degrees	3
	270–360 degrees	4
Thickness	Absent	0
	Thick ≥ 1.5 mm	1
	Thin <1.5 mm	3
Morphology	Indistinguishable	0
	Irregular/Patchy	1
	Continuous	4

*< 7: Dominant intimal; ≥ 7: Dominant non-intimal*.

One of the shortages of the visual systems is the subjectivity between distinct grading scales, which is the notable obstacle in maintaining consistencies. Another considerable defect is the interference by adjacent bony structures, for instance, the skull base around the carotid siphon and the VA (6). Recently, high-resolution MRI (HR-MRI) has been applied to evaluate lesions of intracranial vessel walls (20). By presenting hypointensity, IAC can be detected by HR-MRI. Revealing the ultrastructure of the vessel wall, the HR-MRI enables the neurologists to identify the calcium deposits at different locations (e.g., superficial and deep) and their positional relationship with other plaque components (21). By comparing the autopsy and multi-contrast HR-MRI, Jiang et al. (22) accomplished a remarkable differentiation between lipid core (isointense/hyperintense), fibrous cap (isointense), and calcification (hypointense) on T1 sequence. The combination of HR-MRI and CT will presumably benefit both diagnosis and differentiation of IAC and, in addition, will indicate a necessity for the classification of IAC located at different layers of the vessel wall (intima and media).

## Histological Features of Calcifications

The structure of the wall of the intracranial arteries consists of three layers: the intima (the inner layer), the media (the middle layer), and the adventitia (the outer layer). Different from extracranial arteries which are rich in elastin filaments, intracranial arteries own characteristic features with a denser internal elastic lamina, a thinner media with few elastic fibers, a less abundant adventitia, and an absence of external elastic lamina (23). Vascular calcification, resembling osteogenesis, reflects an osteochondrogenic transformation of smooth muscle cells. Traditionally, calcification is deemed to imply atherosclerosis. Studies in the early 20th century conducted by Mönckeberg confirmed that the origination of medial calcification was independent of atherosclerosis (24). Due to different histological features, calcifications in the intima and the media ought to be discussed separately.

## Intimal Calcification

**Figure 1:**
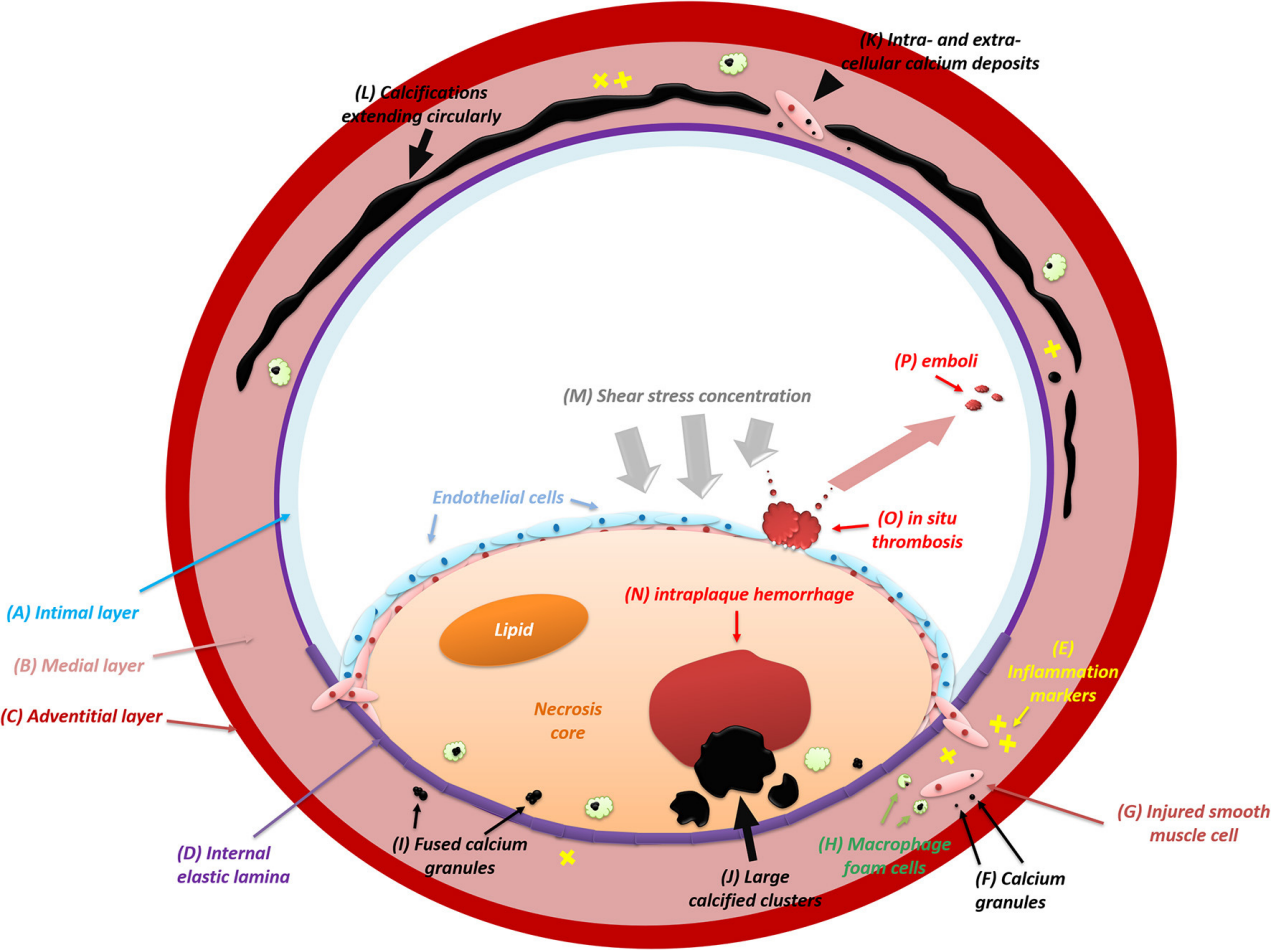
The formation and distribution of intracranial artery calcifications (intimal and medial). IAC mainly involves the intimal layer (A) and the medial layer (B) instead of the adventitial layer (C). During the initial process of atherosclerosis, inflammation (E) infiltrates constantly and intimal calcifications begin to emerge. Usually, calcifications appear as calcium granules (F) within or outside the injured smooth muscle cells (G) and scatter diffusely among extracellular materials. Some of the calcium deposits will be internalized by macrophage foam cells (H). After growing in size with continuous fusion (I) of adjacent granules, calcified deposits will turn into large structures (J). Eventually, with the intima (A) ulcerated by large calcifications, *in situ* thrombosis (O) will adhere to the bare surface of the lesion and release emboli (P) subsequently. In some cases, the shear stress upon the arterial wall will be concentrated (M) on the lesion where large intimal calcifications are present. The elevated shear stress will result in the deformation and rupture of neovessels inside the lesion, leading to intraplaque hemorrhage (N). At first, medial calcifications are irregular calcium deposits (K) distributed intra-cellularly in the vascular muscle cells (G) and extra-cellularly near damaged elastic fibers (D) in the medial layer (B). After confluent extending (L) up to incomplete circumference, medial calcification will distort the architecture of the medial layer and then involve the entire circumference of the vessel wall.

Intimal calcification is characterized by subintimal lipid deposition and macrophage accumulation (25). The intimal layer comprises endothelial cells and the subendothelial connective tissue. During atherosclerosis, inflammation intrudes and the intima becomes thickened gradually with the formation of calcification (26). While absent in primary types, calcium deposits begin to occur as atherosclerosis advances. Figure 1 shows the formation and distribution of intimal calcification. Initially appearing as granules within or outside the injured smooth muscle cells, calcifications diffusely scatter among extracellular materials and some of which are internalized by macrophage foam cells. With a continuous fusion of adjacent granules, calcified granules will turn into larger clusters containing lumps and plates of calcium (24). In some cases, large calcifications may ulcerate the intima, leading to subsequent occlusions (27).

Intimal calcifications frequently occur as thick and patchy clusters (18, 28). Similar to extracranial arteries, histological evidence indicates that intimal calcification frequently [85 (9) and 100% (10)] coexists with intracranial atherosclerosis (ICAS), but its prevalence among all-stage ICAS lesions is not comparatively high [62% (9) and 69% (10)]. This discrepancy could be attributed to the presence of intimal calcification, which is mainly in the progressive ICAS lesions instead of pre-ICAS lesions (10).

Vasa vasorum is a microvasculature network in vessel walls that delivers oxygen and nutrition (29) and transports inflammatory mediators (30–32) that could contribute to atherosclerosis. In 2018, Zheng et al. (33) first reported the association between the density of intraplaque calcification and the presence of adventitial vasa vasorum in *ex vivo* VA specimens, indicating a mutual basis of calcification and ICAS. However, whether intimal or medial calcification is associated with vasa vasorum is yet unknown.

## Medial Calcification

The medial layer of the intracranial vessel wall consists of smooth muscle cells and an elastin-rich extracellular matrix. The normal thickness of the media in the middle cerebral artery (MCA), basilar artery (BA), and VA ranges from 0.15 to 0.19 mm, and it tends to decrease during pathological changes such as atherosclerosis (3). Medial calcifications are deposits of hydroxyapatite with a high degree of crystallization (34). In Figure 1, the formation and extension of medial calcification is briefly drawn. A four-stage criterion is applied to distinguish the calcified lesion in the media layer (35): (1) irregular distribution of intracellular deposits in the vascular muscle cells and in the extra-cellular deposits near damaged elastic fibers in the media (colored in blue or violet) on H&E staining; (2) confluent calcification extending up to incomplete circumference with subendothelial hyperplasia in the intima; (3) calcifications distorting the architecture of media and involving the entire circumference; (4) calcification foci of bone formation.

Focal inflammation also plays a role in medial calcification. A high level of inflammatory markers like C-reactive protein, CD40, and CD154 can be identified in the vicinity of the calcified media (36). The anomalous expression of mineral-regulating proteins may contribute to the process. In patients with chronic kidney disease (CKD), rapid development of medial calcification in the extracranial arteries is observed, partly due to mineral dysregulation stemming from the primary renal disorder (35). Endoplasmic reticulum stress (37) and inflammasome activation (38) may have an impact on the medial calcification, but the key pathogenesis remains hidden.

Medial calcifications appear as thin, continuous, and circular lesions (18, 28). The formation of medial calcification is thought to be independent of atherosclerosis. In intracranial ICAs, the earliest calcifications are located in the medial layer and are unrelated to ICAS (39). More than 60% of non-atherosclerotic medial calcifications in the intracranial ICAs which extend over half of the circumference are irrelevant to the occurrence of the intimal calcification or ICAS while the prevalence of concurrent calcifications is merely 9% (9).

## The Prevalence and Distribution of IAC

The highest prevalence of IAC is found in intracranial ICA (60–80%), followed by that in intracranial VA (17–35%), compared to BA (2.5–7%) and MCA (5%) (40–42). On histology, a low prevalence (3%) of IAC in the major intracranial arteries of Caucasians was reported (43). However, the prevalence of IAC in that of Chinses adults was higher [27.9% in MCA (44) and 39% in the major intracranial arteries (10)].

Different intracranial arteries may have a diverse frequency of IAC. Homburg et al. (45) reported a low prevalence of calcified ICAS lesions (23%) in the distal branches of the circle of Willis. Comparatively, the cavernous and carotid siphon are the most common sites of calcification in ICAs due to their tortuous anatomical configuration (15, 46). Among all segments of VA, the intracranial part is most frequently affected by calcification (40). Left intracranial VA is found more frequently calcified than the right, and most of the IACs in the vertebrobasilar arteries are focal lesions (47), but the mechanism of the left-and-right difference is unclear.

While intimal calcifications tend to occur in all major intracranial arteries (ICA, MCA, VA, and BA) and are always concurrent with ICAS (9, 10), medial calcification is more predominantly present in ICA (9) and VA (10). Histological findings demonstrated that medial calcification contributed more to the total calcified cross-sectional surface area of the carotid artery than the intimal calcification (79 vs. 14%) (9), which indicates their difference in the imaging feature (cluster vs. circular). However, it was also identified that 36% of the medial calcification also had a maximally affected circumference of <50%, meaning a potential cluster-like pattern (9). As a result, the overall calcification on CT scan might not serve as an accurate proxy of atherosclerosis.

## Clinical Relevance of IAC

### Risk Factors

Age (48–50) is an independent risk factor for IAC. In 2005, a CT-based study consisting of 490 consecutive cases demonstrated a high prevalence of calcification (69.4%), among which patients with IAC were significantly older (40). Diabetes (51–53) and CKD (54, 55) are two other major risk factors. As for gender difference, women are found with milder calcification than men (12, 56), but there is a contradictory finding, too (57). Opposite findings were also reported about hypertension (58, 59). One hypothesis suggests that IAC- induced arterial stiffening may act as a cause for the elevated pulse pressure.

Although intimal calcification and medial calcification share mutual risk factors, such as age and higher pulse pressure (39, 60, 61), there are some differences in other traditional risk factors (Table 2). After differentiating into different IAC patterns (intimal and medial), male gender and smoking were found to be independently associated with intimal calcification, while aging and diabetes mellitus were more related to medial calcification (60, 64).

**Table 2:** Comparison of risk factors between intimal and medial calcification (intracranial and periphery arteries).

**Study authors and year**	**Subjects**	**Sample size**	**Risk factors**	
			**Intimal calcification**	**Medial calcification**
Vos et al. (60)	Intracranial ICAs of patients with acute ischemic stroke who received CT scans	1,132	Male, smoking, hypertension	Age, diabetes mellitus, previous vascular disease
Compagne et al. (62)	Intracranial ICAs of patients who received CT scans and underwent thrombectomy	344	Male, smoking, pre-stroke with mRS ≤ 2	Age, atrial fibrillation, diabetes mellitus, myocardial infarction, hypertension
Golüke et al. (61)	Intracranial ICAs of patients with mixed dementia who received CT scans	1,992	Male, hypertension, smoking, myocardial infarction	Diabetes mellitus
Zwakenberg et al. (63)	Femoral arteries of patients who received CT scans	718	Smoking, history of peripheral arterial disease, higher osteonectin level	Age, diabetes, HbA1c, higher ankle brachial index (ABI), higher dp-ucMGP level

### Plaque Vulnerability

The association of IAC with plaque vulnerability remains controversial, which is partially due to the unclassified calcification patterns. A CT study showed that asymptomatic MCA plaques had a higher frequency of IAC compared to symptomatic MCA plaques (8), implying that IAC has a protective effect on the intracranial arteries. On the other hand, micro embolic signals in the intracranial ICA ipsilateral to acute MCA infarct were more frequently detected by transcranial Doppler (TCD) in patients with a higher extent of calcification (widest arc of IAC ≥ 90°) than those with the lower extent of calcification (13). However, in the TCD-based study (13), patients were diagnosed with concomitant MCA stenosis; hence the embolic signals in the temporal window originated exactly from the calcified lesion of ICA was indistinguishable. Moreover, latent carotid calcification is also related to the risk of cardio-aortic embolism (65), which can be a major interference in emboli detection.

**Figure 2:**
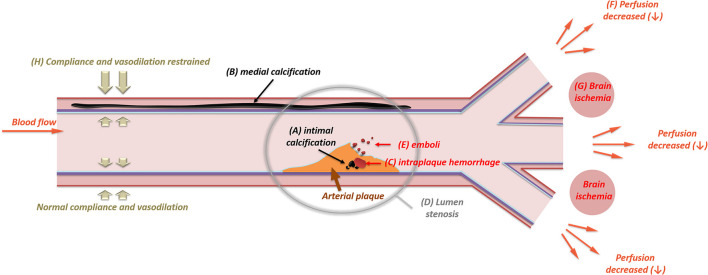
The impact of intracranial artery calcifications (intimal and medial) on ischemic stroke. The presence of intimal calcification (A) may induce intraplaque hemorrhage (C). Intraplaque hemorrhage is one of the major causes for the rapid expanding of arterial plaque, which will then result in luminal stenosis (D). In some cases, the intima will be ulcerated by large calcification, leading to distal embolism (E). With an increase in the stenotic degree, the perfusion of the downward vascular territory will decrease (F), leading to brain ischemia (G) in the border zone of adjacent vascular branches. Unlike intimal calcifications, medial calcification (B) mainly causes arterial stiffness. The stiffened arterial wall will have limited compliance and vasodilation (H), which can be measured through carotid-femoral pulse wave velocity, systolic flow velocity, and pulsatile index.

So far, knowledge of the plaque vulnerability in different IAC patterns is still limited. In contrast, the link between calcification and plaque vulnerability in the coronary artery and the carotid artery is more thoroughly studied, which may serve as a reference. In coronary atherosclerosis, the superficial calcified nodule is an independent risk factor for plaque rupture (66). One of the most vulnerable sites is the junction between calcification and soft plaque within the fibrous cap, where the shear stress tends to increase and eventually causes rupture (67). In the carotid artery, superficial calcification in the plaque was related to intraplaque hemorrhage (IPH) (7, 68), a strong predictor for ipsilateral cerebrovascular events (69–71). One possible explanation about the pathophysiology of calcification related to IPH is the change of focal pressure. Due to the presence of calcification, the shear stress of the blood flow is increased and concentrated on the plaque surface (Figure 2) (72, 73), which may cause the deformation and rupture of neovessels inside the plaque (74). Compared to superficial calcifications, deep calcium tends to show irrelevance to IPH in the carotid artery (7). Since “deep” calcified deposits are more possibly located in the medial (or adventitia) layer, medial calcification appears to be unrelated to plaque vulnerability.

### Hemodynamic Impact

Intimal calcification is related to atherosclerotic luminal stenosis (10), a pivotal cause of territorial hypoperfusion and artery-to-artery embolism (Figure 2) (75). Previous studies have revealed the correlation between luminal stenosis and the severity (thickness and circularity) of calcification in the intracranial ICA (76, 77). In contrast, medial calcification seems to be more associated with vascular remodeling than luminal narrowing (78) in the vertebral artery and coronary artery (79). No significant correlation between medial calcification and luminal stenosis has been established yet (10). Medial calcification is thought to affect arterial stiffening, resulting in compliance deterioration and vasodilation limitation (Figure 2) (80). However, in previous studies about intracranial arteries, the IAC pattern was seldom categorized. Notwithstanding, the correlation of medial calcification is still partially deducible. Patients with IAC have higher carotid-femoral pulse wave velocity (81). Additionally, in the MCA and VA, heavier IAC may lead to elevated systolic flow velocity and pulsatile index, which indicate high resistance within the cerebral vasculature (82). IAC may also protect the artery from vasospasm under the circumstance of aneurysmal subarachnoid hemorrhage (83).

Arterial wall stiffening caused by calcification is an independent risk factor for all-cause mortality (84). Compagne et al. reported a trend toward worse outcomes in patients with medial calcification who would benefit more after endovascular thrombectomy compared to intimal calcification (62). Severe calcification is associated with incomplete arterial revascularization after mechanical thrombectomy (85) and prolonged procedural times during endovascular therapy (86). Besides the luminal stenosis caused by IAC, arterial stiffness is also a detrimental factor in the process of endovascular thrombectomy that blocks the extraction of thrombus and therefore increases the passes of the retriever. Furthermore, patients with heavier IAC burden in either the anterior (87) or posterior (88) circulation tend to have poor clinical outcomes after endovascular thrombectomy. It is conceivable that the loss of elasticity may decrease the microvascular cerebral perfusion, resulting in arterial flow stasis and diffuse thrombogenesis. In some diseases, IAC is found protective. Patients with IAC tend to suffer less often from arterial dissection than those without arterial dissection (60). Whether stiffening acts as a reason is unknown.

### IAC in Cerebrovascular Events

Intracranial artery calcification has drawn attention as an independent risk factor for stroke (4). Chen et al. (41) first described a high prevalence of IAC in Chinese adults with ischemic stroke. An upgoing incidence of early vascular events lies toward the severity of IAC in ischemic stroke or transient ischemic attack (89). Patients with heavier calcification are at a higher risk of suffering from large cerebral artery occlusion (90) and recurrent ischemic stroke (91, 92). However, despite the fact that ischemic stroke is a condition with divergent causes including large artery atherosclerosis, cardiovascular embolism, small vessel disease, and other determined or undetermined etiology, prior studies mostly focused on the association of IAC with all causes of stroke. The Rotterdam study revealed the association of IAC with stroke (4), but many of the stroke events were in the vascular territories that were separated from IAC and were led by other vascular disorders (93). In a prospective study on patients with ischemic stroke, the presence and score of IAC were found to be associated with recurrent stroke events (94). However, patients with vascular events had more intracranial atherosclerotic plaques, which may also account for infarction. The coexistence of IAC, atherosclerotic plaque, and luminal narrowing makes it difficult to be determined. In contrast to these findings, a protective effect of calcification was reported (8), but further studies with larger sample size and more specific IAC classifications are needed.

Intracranial artery calcification also has an impact on lacunar infarcts and white matter hyperintensity (42, 95), presumably resulting from an injured vascular tone and vasodilatory after endothelial impairment (96, 97). Erbay et al. (98) reported a weak link of IAC to white matter hyperintensities after adjusting for age. In terms of cerebral hemorrhage, significant expansion of hematoma was observed in the presence of IAC (99). Evidence showed that IAC was a predictor for microbleeds (52, 100) and hemorrhagic transformation after intravenous thrombolysis (101, 102). Medial calcification seemed more correlated to hemorrhagic complications after intravenous thrombolytic therapy (103). The increased frequency of microbleeds in patients with IAC may be due to microvascular impairment (104). Recently, a possible link was reported between Fahr's disease (familial idiopathic basal ganglia calcification) and calcified small vessels that supply the basal ganglia (105). It is conceivable that small vessel impairment may be attributed to IAC since it systemically affects multiple vessel beds.

The pathophysiology of calcification leading to cerebrovascular disease has not been fully elucidated. Intimal calcification often coexists with atherosclerosis, during which endothelial function will be impaired (106) and the permeability of the brain-blood barrier may increase subsequently. Medial calcification can lead to arterial stiffness by damaging the elastic fiber around the internal elastic lamina of the medial layer. With deterioration in compliance, distal cerebral microvascular with increased blood pressure will be vulnerable to rupture.

### Intracranial Artery Calcification With Cognitive Disorder

The association between cognitive disorder and IAC has been studied in recent years. A cross-sectional study with 1992 recruited patients who were diagnosed with different types of cognitive dysfunction (107) revealed a high incidence (about 95%) of intracranial internal carotid artery calcification (61). Cognitive impairment had been observed among patients diagnosed with IAC and concurrent conditions, such as chronic hypoparathyroidism (108) and hemodialysis (109) that could directly result in irregular serum calcium. Among patients without such conditions, the risk of dementia could also rise with larger IAC volume (110), despite the influence of stroke. Additionally, patients with larger IAC volume (111) or area (112) were found to perform worse during neuropsychological assessments. However, the link between IAC and type of dementia or cognitive disorder turned uncertain after adjustment for age and gender. Similar findings were reported in the severity of mixed dementia, which was identified as “not associated” with IAC after additional adjustment for cardiovascular risk (61). The correlation of IAC to cognitive dysfunction remained unclear. One possible cause could be the focus only on IAC in the intracranial ICA, since the prevalence of IAC in other vessel beds is comparatively much lower.

Due to its long preclinical stage in which subtle cognitive deficits could only be revealed by dedicated neuropsychological tests (113), dementia or cognitive decline is always barely noticed by patients until the emergence of evident symptoms. Whether IAC can serve as a bio-marker for early screening of dementia or cognitive decline might depend on further studies including a large number of recruited patients with more intracranial arteries.

## Discussion

Intracranial artery calcification includes two major types: intimal calcification and medial calcification, in which the histopathological features are different from each other. Table 3 shows the main difference between intimal calcification and medial calcification. Intimal calcification is more related to focal atherosclerotic lesion while medial calcification tends to spread over the medial layer. Non-atherosclerotic medial calcification is predominantly present in both the intracranial ICA and VA while intimal calcification can occur in all major cerebral arteries. Due to different histological features of intimal and medial calcification, the traditional quantitative measurement could be insufficient to reflect on accurate clinical information, indicating a demand for new measurements by CT or MRI.

**Table 3:** Summary of the difference between intimal calcification and medial calcification.

**IAC pattern**	**Pathological feature**		**Risk factor (difference)**	**Clinical impact**	
	**Formation**	**Morphology**		**Stroke mechanism**	**Clinical prognosis**
Intimal calcification	(1) Related to atherosclerosis, often in advanced stages; (2) Inflammation associated; (3) Granules initially, fuse into large lumps and plates of calcium (4) May ulcerate the intima	Thick / patchy clusters	Male gender; Smoking	(1) Elevated shear stress causing IPH; (2) Hypoperfusion (luminal stenosis)	With lower incidence of hemorrhage after intravenous-thrombolysis
Medial calcification	(1) Irrelevant to atherosclerosis; (2) Inflammation associated; (3) Four-stage formation: from deposits to confluent calcification involving the entire circumference	Thin, often in a circular pattern	Aging; Diabetes mellitus; Chronic kidney disease;	(1) Arterial stiffness; (2) Possibly worse perfusion in microvascular beds	With a trend toward worse outcome which may improve more after endovascular treatment

Intimal calcification differs from medial calcification in risk factors, the association with plaque vulnerability, and the hemodynamic impact. It plays a critical role in IPH and luminal stenosis while medial calcification is more connected to arterial stiffness and vasodilation. The causal correlation of calcification with infarction and the influence of separate IAC patterns are unknown,. IAC is considered a risk factor for cerebral small vessel disease, most likely due to endothelial dysfunction. The pathophysiology underlying the IAC-inducing stroke is still unclear. Further histological, imaging, and clinical evidence that are based on different IAC subtypes are required in future studies.

## Author Contributions

HD and XC contributed to the conception and design of the study. WY and XC organized the database. HD wrote the first draft of the manuscript. All authors contributed to manuscript revision, read, and approved the submitted version.

## Conflict of Interest

The authors declare that the research was conducted in the absence of any commercial or financial relationships that could be construed as a potential conflict of interest.

## Publisher's Note

All claims expressed in this article are solely those of the authors and do not necessarily represent those of their affiliated organizations, or those of the publisher, the editors and the reviewers. Any product that may be evaluated in this article, or claim that may be made by its manufacturer, is not guaranteed or endorsed by the publisher.
